# Supplementary material for: Time-series transcriptome analysis identified differentially expressed genes in broiler chicken infected with mixed Eimeria species
Source: Front Genet. 2022 Aug 8;13:886781. doi: 10.3389/fgene.2022.886781 (PMC9393255; doi:10.3389/fgene.2022.886781)
Supplement: Supplementary file 2 [file DataSheet1.ZIP › 4dpi_KEGG.Gsea.1625100298584/KEGG_GLUTATHIONE_METABOLISM.html]

Details for gene set KEGG\_GLUTATHIONE\_METABOLISM[GSEA]

|  || Dataset | TMM\_4dpi\_gct\_format\_4dpi\_gct\_format.Class\_4dpi.cls #PC\_versus\_NC.Class\_4dpi.cls #PC\_versus\_NC\_repos |
| Phenotype | Class\_4dpi.cls#PC\_versus\_NC\_repos |
| Upregulated in class | 0 |
| GeneSet | KEGG\_GLUTATHIONE\_METABOLISM |
| Enrichment Score (ES) | -0.4599022 |
| Normalized Enrichment Score (NES) | -1.5539463 |
| Nominal p-value | 0.023596035 |
| FDR q-value | 0.17705254 |
| FWER p-Value | 0.8424 |
Table: GSEA Results Summary

  

Fig 1: Enrichment plot: KEGG\_GLUTATHIONE\_METABOLISM      
 Profile of the Running ES Score & Positions of GeneSet Members on the Rank Ordered List

  

| SYMBOL | TITLE | RANK IN GENE LIST | RANK METRIC SCORE | RUNNING ES | CORE ENRICHMENT || 1 | RRM2 | na | 1152 | 0.618 | -0.0533 | No |
| 2 | GSTT1 | na | 1493 | 0.521 | -0.0456 | No |
| 3 | GPX7 | na | 1895 | 0.440 | -0.0486 | No |
| 4 | RRM1 | na | 2238 | 0.383 | -0.0506 | No |
| 5 | GSTM2 | na | 2462 | 0.353 | -0.0447 | No |
| 6 | TXNDC12 | na | 2919 | 0.294 | -0.0624 | No |
| 7 | GGT7 | na | 3383 | 0.234 | -0.0848 | No |
| 8 | ODC1 | na | 3763 | 0.191 | -0.1032 | No |
| 9 | PGD | na | 4349 | 0.138 | -0.1425 | No |
| 10 | GPX3 | na | 4629 | 0.112 | -0.1580 | No |
| 11 | GGCT | na | 4993 | 0.079 | -0.1828 | No |
| 12 | IDH1 | na | 5624 | 0.024 | -0.2338 | No |
| 13 | GCLM | na | 5819 | 0.008 | -0.2494 | No |
| 14 | MGST2 | na | 5918 | -0.001 | -0.2576 | No |
| 15 | GGT5 | na | 7285 | -0.116 | -0.3636 | No |
| 16 | RRM2B | na | 7489 | -0.134 | -0.3712 | No |
| 17 | GSTZ1 | na | 7777 | -0.159 | -0.3841 | No |
| 18 | GPX4 | na | 7910 | -0.171 | -0.3833 | No |
| 19 | GSR | na | 8688 | -0.250 | -0.4309 | Yes |
| 20 | GSS | na | 8801 | -0.261 | -0.4221 | Yes |
| 21 | GSTK1 | na | 9125 | -0.299 | -0.4284 | Yes |
| 22 | IDH2 | na | 9421 | -0.339 | -0.4295 | Yes |
| 23 | GSTO2 | na | 9462 | -0.343 | -0.4091 | Yes |
| 24 | MGST3 | na | 10072 | -0.424 | -0.4305 | Yes |
| 25 | LAP3 | na | 10138 | -0.435 | -0.4057 | Yes |
| 26 | SRM | na | 10221 | -0.448 | -0.3815 | Yes |
| 27 | ANPEP | na | 10739 | -0.547 | -0.3868 | Yes |
| 28 | GSTA2 | na | 10745 | -0.548 | -0.3492 | Yes |
| 29 | GPX1 | na | 10839 | -0.569 | -0.3175 | Yes |
| 30 | MGST1 | na | 11272 | -0.692 | -0.3056 | Yes |
| 31 | GSTA3 | na | 11653 | -0.916 | -0.2738 | Yes |
| 32 | GCLC | na | 11745 | -1.013 | -0.2111 | Yes |
| 33 | SMS | na | 11937 | -1.476 | -0.1247 | Yes |
| 34 | GSTA4 | na | 11979 | -1.885 | 0.0026 | Yes |
Table: GSEA details [plain text format]

  

Fig 2: KEGG\_GLUTATHIONE\_METABOLISM      
 Blue-Pink O' Gram in the Space of the Analyzed GeneSet

  

Fig 3: KEGG\_GLUTATHIONE\_METABOLISM: Random ES distribution      
 Gene set null distribution of ES for **KEGG\_GLUTATHIONE\_METABOLISM**
